# Supplementary material for: Theoretical Study on the Electronic Structure and Magnetic Properties Regulation of Janus Structure of M’MCO2 2D MXenes
Source: Nanomaterials (Basel). 2022 Feb 6;12(3):556. doi: 10.3390/nano12030556 (PMC8838217; doi:10.3390/nano12030556)
Supplement: Supplementary file 1 [file nanomaterials-12-00556-s001.zip › nanomaterials-1569494-supplementary.pdf]

Supplementary Materials

Theoretical Study on the Electronic Structure and Magnetic Properties Regulation of Janus Structure of M'MCO$_2$ 2D MXenes

Panpan Gao [1,2], Minhui Song [1,2], Xiaoxu Wang [1,2], Qing Liu [1,2,3], Shizhen He [1,2], Ye Su [1,2,*] and Ping Qian [1,2,*]

1 Beijing Advanced Innovation Center for Materials Genome Engineering, University of Science and
Technology Beijing, Beijing 100083, China; gaopanpan@ustb.edu.cn (P.G.); mhuisong@163.com (M.S.); wangxx@dp.tech (X.W.); liuqing6903@163.com (Q.L.); hsz0430@163.com (S.H.)

2 School of Mathematics and Physics, University of Science and Technology Beijing, Beijing 100083, China

3 Department of Physics, National University of Singapore, Singapore 117551, Singapore

* Correspondence: suyechina@163.com (Y.S.); qianping@ustb.edu.cn (P.Q.)

Basic information of lattice parameters after structural optimization

Table S1 the lattice parameters of $VCrCO_2$

	Initial	NM	FM	AFM1	AFM2	AFM3
Symmetry Group	P3m1 (C_{3V-1})	P3m1 (C_{3V-1})	P3m1 (C_{3V-1})	Cm (C_{s-3})	P3m1 (C_{3V-1})	P3m1 (C_{3V-1})
IT Number	156	156	156	8	156	156
a/Å	2.88	2.85	2.95	2.96	5.87	5.88
b/Å	2.88	2.85	2.95	2.96	2.95	2.96
c/Å	21.81	21.81	21.81	21.81	21.81	21.81
α	90.00°	90.00°	90.00°	90.00°	90.00°	90.00°
β	90.00°	90.00°	90.00°	90.00°	90.00°	90.00°
γ	120.00°	120.00°	120.00°	119.73°	120.24°	120.25°

Table S2 the lattice parameters of $VMnCO_2$

	Initial	NM	FM	AFM1	AFM2	AFM3
Symmetry Group	P3m1 (C_{3V-1})	P3m1 (C_{3V-1})	Cm (C_{s-3})	Cm (C_{s-3})	Pc (C_{s-2})	Cm (C_{s-3})
IT Number	156	156	8	8	7	8
a/Å	2.89	2.87	2.96	3.01	5.92	5.90
b/Å	2.89	2.87	2.96	2.97	2.96	2.99
c/Å	21.81	21.81	21.81	21.81	21.81	21.81
α	90.00°	90.00°	90.00°	90.00°	90.00°	90.00°
β	90.00°	90.00°	90.00°	90.00°	90.00°	90.00°
γ	120.00°	120.00°	119.41°	120.55°	120.24°	120.40°

Table S3 the lattice parameters of $CrMnCO_2$

	Initial	NM	FM	AFM1	AFM2	AFM3
Symmetry Group	P3m1 (C_{3V-1})	P3m1 (C_{3V-1})	P3m1 (C_{3V-1})	P3m1 (C_{3V-1})	P3m1 (C_{3V-1})	Cm (C_{s-3})
IT Number	156	156	156	156	156	8
a/Å	2.66	2.66	2.93	2.88	5.76	6.61
b/Å	2.66	2.66	2.93	2.88	2.91	3.33
c/Å	21.81	21.81	21.81	21.81	21.81	21.81
α	90.00°	90.00°	90.00°	90.00°	90.00°	90.00°
β	90.00°	90.00°	90.00°	90.00°	90.00°	90.00°
γ	120.00°	120.00°	120.00°	120.00°	120.41°	120.37°

Table S4 the lattice parameters of V_2CO_2

	Initial	NM	FM	AFM1	AFM2	AFM3
Symmetry Group	$P\bar{3}m1$ (D_{3d-3})	$P\bar{3}m1$ (D_{3d-3})	C2/m (C_{2h-3})	C2/m (C_{2h-3})	C2/m (C_{2h-3})	P2/c (C_{2h-4})
IT Number	164	164	12	12	12	13
a/Å	2.88	2.88	2.95	3.06	5.98	5.99
b/Å	2.88	2.88	2.95	2.96	2.94	2.95
c/Å	21.81	21.81	21.81	21.81	21.81	21.81
α	90.00°	90.00°	90.00°	90.00°	90.00°	90.00°
β	90.00°	90.00°	90.00°	90.00°	90.00°	90.00°
γ	120.00°	120.00°	117.78°	121.17°	119.47°	119.52°

Table S5 the lattice parameters of Cr_2CO_2

	Initial	NM	FM	AFM1	AFM2	AFM3
Symmetry Group	$P\bar{3}m1$ (D_{3d-3})	$P\bar{3}m1$ (D_{3d-3})	$P\bar{3}m1$ (D_{3d-3})	$P\bar{3}m1$ (D_{3d-3})	$P\bar{3}m1$ (D_{3d-3})	C2/m (C_{2h-3})
IT Number	164	164	164	164	164	12
a/Å	2.68	2.68	2.89	2.76	5.64	5.87
b/Å	2.68	2.68	2.89	2.76	2.83	2.88
c/Å	21.81	21.81	21.81	21.81	21.81	21.81
α	90.00°	90.00°	90.00°	90.00°	90.00°	90.00°
β	90.00°	90.00°	90.00°	90.00°	90.00°	90.00°
γ	120.00°	120.00°	120.00°	120.05°	120.08°	119.43°

Table S6 the lattice parameters of Mn_2CO_2

	Initial	NM	FM	AFM1	AFM2	AFM3
Symmetry Group	$P\bar{3}m1$ (D_{3d-3})	$P\bar{3}m1$ (D_{3d-3})	$P\bar{3}m1$ (D_{3d-3})	$P\bar{3}m1$ (D_{3d-3})	$P\bar{3}m1$ (D_{3d-3})	$P\bar{3}m1$ (D_{3d-3})
IT Number	164	164	164	164	164	164
a/Å	2.87	2.73	2.95	2.97	5.85	5.87
b/Å	2.87	2.73	2.95	2.97	2.97	2.93
c/Å	21.81	21.81	21.81	21.81	21.81	21.81
α	90.00°	90.00°	90.00°	90.00°	90.00°	90.00°
β	90.00°	90.00°	90.00°	90.00°	90.00°	90.00°
γ	120.00°	120.00°	120.00°	120.00°	120.42°	119.93°

Magnetic moment information

The unit of atomic magnetic moment is μB/atom, and the unit of total magnetic moment is μB/formula unit.

Table S7 the magnetic moment of $VCrCO_2$

	FM	AFM1	AFM2	AFM3
V	0.762	0.594	0.307	0.306
Cr	2.801	2.786	2.785	2.786
C	0.259	0.018	0.015	0.015
O	0.118	0.099	0.026	0.026
total	3.068	2.124	0.001	0.003

Table S8 the magnetic moment of $VMnCO_2$

	FM	AFM1	AFM2	AFM3
V	1.039	1.192	1.145	0.933
Mn	3.379	3.371	3.318	3.277
C	0.223	0.099	0.025	0.048
O	0.076	0.063	0.026	0.018
total	4.042	2.059	0.000	0.000

Table S9 the magnetic moment of $CrMnCO_2$

	FM	AFM1	AFM2	AFM3
Cr	2.585	2.457	2.652	3.071
Mn	3.706	3.686	3.59	4.333
C	0.552	0.035	0.023	0.199
O	0.264	0.141	0.071	0.049
total	5.212	1.112	0.001	0.000

Table S10 the magnetic moment of V_2CO_2

	FM	AFM1	AFM2	AFM3
V	1.115	1.116	1.052	0.978
C	0.136	0.000	0.000	0.042

O	0.056	0.053	0.032	0.031
total	1.982	0.000	0.000	0.000

Table S11 the magnetic moment of Cr_2CO_2

	FM	AFM1	AFM2	AFM3
Cr	2.582	2.321	2.430	2.460
C	0.516	0.003	0.024	0.270
O	0.232	0.104	0.077	0.077
total	4.184	0.034	0.386	0.019

Table S12 the magnetic moment of Mn_2CO_2

	FM	AFM1	AFM2	AFM3
Mn	3.332	3.413	3.385	3.146
C	0.311	0.000	0.000	0.073
O	0.127	0.054	0.022	0.031
total	6.097	0.000	0.000	0.000
